# Sleeve resection for bronchial carcinoid tumour in two children under six years old

**DOI:** 10.1186/s12957-016-0870-0

**Published:** 2016-04-14

**Authors:** Basak Erginel, Berker Ozkan, Feryal Gun Soysal, Alaaddin Celik, Tansu Salman, Alper Toker

**Affiliations:** Department of Pediatric Surgery, Istanbul Faculty of Medicine, Istanbul University, Millet caddesi, Capa, 34093/Fatih, Istanbul, Turkey; Department of Thoracic Surgery, Istanbul Faculty of Medicine, Istanbul University, Istanbul, Turkey

**Keywords:** Bronchial carcinoid tumour, Sleeve resection, Paediatric

## Abstract

**Background:**

Paediatric tracheobronchial tumours are very rare, and pneumonectomy and lobectomy procedures are rarely indicated due to their surgical difficulties and high sequelae. Bronchoplastic techniques preserving lung parenchyma allow the resection and reconstruction of the main bronchi and carina.

**Case Presentation:**

Here, we present a 6-year-old boy suffering from a carcinoid tumour of the right main bronchus which was successfully managed with a right upper sleeve lobectomy and a 4-year-old girl with an endobronchial carcinoid tumour narrowing the left main bronchus that received a sleeve resection of that bronchus.

**Conclusion:**

Bronchoplastic techniques are widely used in adults, can be very successful in paediatric patients where the preservation of the lung parenchyma is more important.

## Background

The bronchial carcinoid (BC) tumour is a neuroendocrine tumour of the lung presenting in 1–2 % of all lung neoplasia cases; however, BC tumours are very rare in the paediatric population. These tumours, which invade the airways causing a cough, haemoptysis and recurrent pulmonary infection, are categorised as typical carcinoid (TC) or atypical carcinoid (AC) according to their histopathologies [1]. Moreover, total surgical excision is important for a good prognosis. Recently, bronchoplastic techniques have been used to treat BC tumours in the airways of adults, and these techniques have also been suggested for paediatric patients, where the residual capacity after surgery is much more important [2]. However, the literature includes few reports of sleeve resections for BC tumours in the first decade of life [3].

## Case presentation

### Case 1

**Fig. 1:**
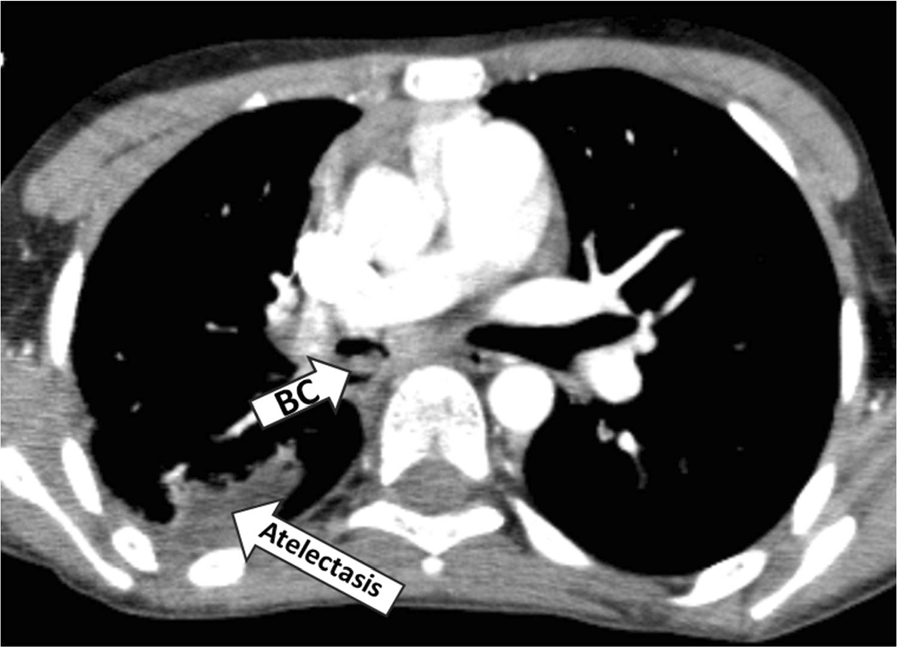
Thoracic computed tomography of case 1, revealing a solid 18 × 5 mm mass in the right main bronchus (BC) and post-obstructive atelectasis in the right upper lobe

A 6-year-old boy presented at the paediatric intensive care unit of our hospital with acute respiratory failure, and his chest x-ray revealed a closed right hemithorax. A rigid bronchoscopic examination was done based on the suspicion of a foreign body, revealing an endobronchial mass on the right main bronchus. The histological examination of the biopsy was consistent with BC. Furthermore, his thoracic computed tomography (CT) revealed a solid 18 × 5 mm mass in the right main bronchus leading to the obstruction (Fig. 1).

**Fig. 2:**
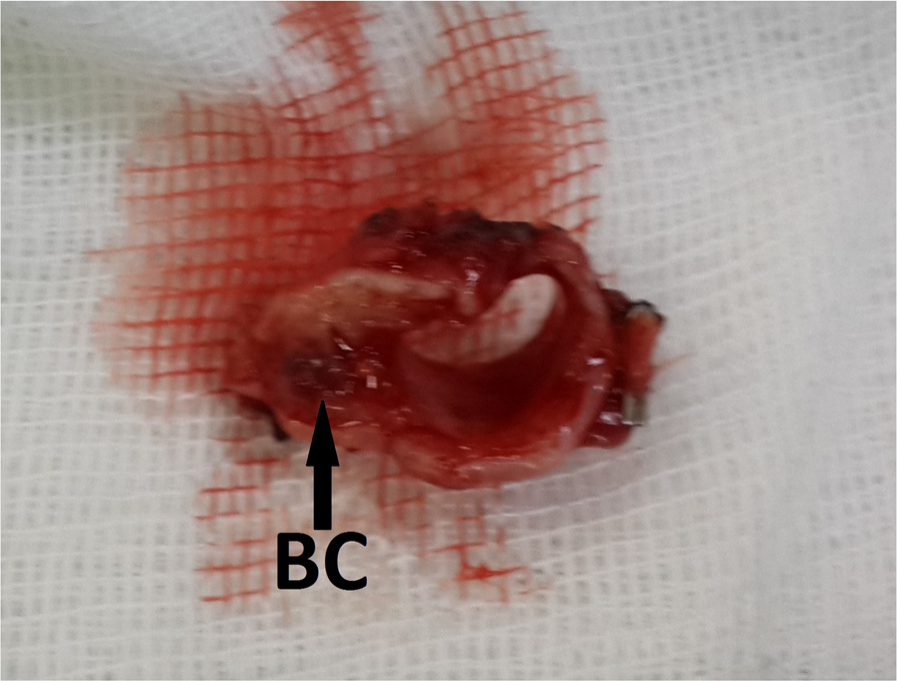
Resected segment of the right main bronchus in case 1 (*BC* bronchial carcinoid tumour)

**Fig. 3:**
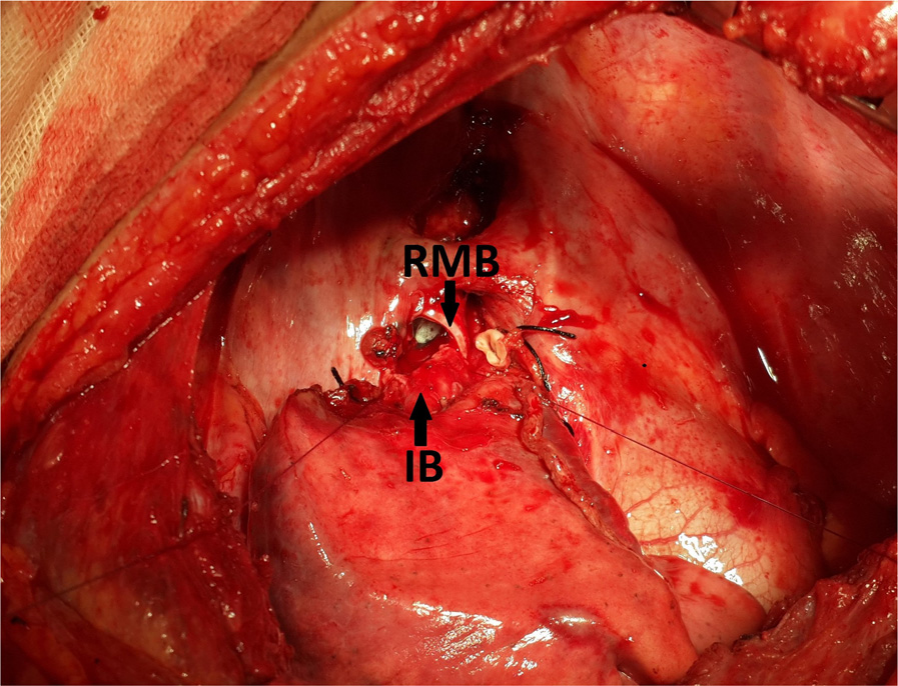
Anastomosis of the intermediate bronchus to the right main bronchus at the level of the carina (*RMB* right main bronchus, *IB* intermediate bronchus)

To treat this patient, a right upper sleeve resection was performed under single-lung ventilation. The right main bronchus was dissected 0.5 cm distally from the carina, and an en bloc resection of the tumour was performed (Fig. 2). The surgical margins of the right main bronchus and intermediate bronchus were reported to be tumour-free in the evaluation of the frozen sections. The intermediate bronchus was then anastomosed to the right main bronchus at the level of the carina, with a continuous technique 4/0 PDS (Polydioxanone) suture in the membranous part and a separating technique in the cartilaginous part (Fig. 3).

Interlobar, hilar and mediastinal lymph node dissections were also conducted, and the pathological report confirmed T3N0M0 (stage IIB) typical BC with tumour-free margins. No short-term complications occurred; therefore, the patient was discharged on postoperative day 7 and has been under follow-up for 2 years.

### Case 2

A 4-year-old girl was admitted to our department with complaints of coughing, wheezing and recurrent pneumonia, and her chest x-ray revealed left atelectasis. Her thoracic CT revealed an endobronchial mass obstructing the left main bronchus, and rigid bronchoscopy showed an endobronchial mass narrowing the left main bronchus.

Under single-lung ventilation, this patient was treated with a sleeve resection of the left main bronchus and an end-to-end anastomosis with a continuous suture using 4/0 PDS in the membranous part and separate sutures for the cartilaginous part. A routine lymph node dissection was also performed, and the specimen was identified as T3N0M0 (stage IIB) typical BC with tumour-free margins. This patient was discharged on postoperative day 8 and has been under follow-up observation for 4 years without any recurrence.

### Discussion

Primary pulmonary tumours in childhood are very rare, and of these rare tumours in the paediatric population, BC tumours (low-grade malignant neoplasms originating from neuroendocrine cells) are the most common [4]. Although pneumonectomies and lobectomies have typically been performed for bronchial tumours, the use of parenchyma-saving procedures, such as sleeve resections and bronchoplasties, has recently increased. It is extremely important to preserve the parenchyma to avoid the morbidity and sequelae that can occur throughout a patient’s lifespan. Moreover, the aim of any surgical procedure is a resection with tumour-free margins while preserving the maximum amount of parenchyma. Only a few paediatric cases of BC treated with sleeve resections have been reported in the literature.

In the adult population, tracheobronchial sleeve resection is the treatment of choice for BC tumours for the best short- and long-term outcomes [5]. Gaissert et al. [2] proposed the procedure in the paediatric population and presented a series of 12 patients (aged 8–19 years old), including four with BC. Later, Toker et al. [3] reported two sleeve resections in patients under 10 years old for carcinoid tumours. Additionally, Rizzardi et al. [6] reported 15 children who underwent 10 parenchyma-saving procedures (five sleeve lobectomies, three sleeve resections of the main bronchus and two bronchoplasties).

Recently, there have been case reports of sleeve resections for other endobronchial tumours; for example, Pan et al. [7] reported the case of a 4-year-old boy with a myofibroblastic tumour who had a left inferior sleeve lobectomy. Additionally, Wildbrett et al. [8] reported the case of a 6-year-old boy with mucoepidermoid carcinoma who underwent a right upper sleeve resection, while de Agustín et al. [9] presented the case of a 5-year-old girl who underwent sleeve resection for a mucoepidermoid carcinoma on the left main bronchus.

Sleeve bronchoplasty techniques are complex surgical procedures, especially in children, whose bronchial systems are much smaller and more delicate. In this case, we performed a bronchoscopy preoperatively and conducted perioperative frozen section studies and lymph node sampling in both of our cases (these are important to confirm tumour-free margins). Extracorporeal membrane oxygenation (ECMO) or similar treatments was not needed during or after the bronchial sleeve resection*.* Continuous suture 4/0 PDS was used in the membranous area, and a separating technique was used for the cartilaginous area in order to avoid anastomotic stricture during the follow-up period. For the postoperative follow-up of our patients, we used a CT scan, while the follow-up periods of our patients were 2 and 4 years without recurrence or any other complications.

As in our second case, an isolated sleeve of the main bronchus is a good choice for treatment in endobronchial main bronchi tumours in paediatric as well as adult patients in order to preserve the whole lung parenchyma [10]. Moreover, lymph node dissection for accurate staging is a major component of the surgical management of BCs, since these tumours have a greater than 10 % risk of lymph node metastasis [11]. In both of our cases, we performed lymph node dissections.

Overall, Yu et al. [12] presented the largest comparison evaluating the efficacy of this treatment between paediatric and adult cases and found that sleeve resection also has an excellent prognosis in the paediatric population.

In the case of the failure of the anastomosis, the risk of a bronchopleural fistula and the aspiration of the empyema to the healthy lung should be kept in mind; therefore, in suspicious cases, the anastomosis should be rapidly checked via bronchoscopy. Previously, routine bronchoscopies were recommended for the control of each sleeve’s bronchoplasty; however, they are not used now, although we do advise beginners in such bronchoplastic techniques to use them. Our second recommendation is to avoid excessive dissection for the prevention of major air leakage. Dissections under 5 mm are not recommended.

To the best of our knowledge, these are the first cases of BC tumours treated with sleeve resection in patients under the age of 5 years old. Since no series exists for this aspect of treatment, our case studies should encourage paediatric thoracic surgeons to utilise sleeve resection techniques in patients younger than 5 years old.

## Conclusions

The sleeve lobectomy technique for BC tumors provides a better postoperative period, low postoperative morbidity and higher quality of life in the long term for a growing child. Bronchial reconstructive lung-sparing operations should be considered for appropriate bronchial tumours even in the first 5 years of life.

### Consent

The patients’ parents granted written informed consent for the publication of this manuscript and the accompanying images.
